# Surgically treated cervical cancer in a high-risk group in the era of the 2018 FIGO staging schema: a nationwide study

**DOI:** 10.1038/s41598-023-39014-8

**Published:** 2023-07-25

**Authors:** Shogo Shigeta, Muneaki Shimada, Keita Tsuji, Zen Watanabe, Yasuhito Tanase, Koji Matsuo, Toru Nakanishi, Toshiaki Saito, Daisuke Aoki, Mikio Mikami

**Affiliations:** 1 Department of Obstetrics and Gynecology, Tohoku University School of Medicine, 1-1 Seiryo-Machi, Aoba-Ku, Sendai, Miyagi 980-8574 Japan; 2 Department of Obstetrics and Gynecology, Yokohama City University, Kanagawa, Japan; 3 Department of Gynecology, National Cancer Center Hospital, Tokyo, Japan; 4 Division of Gynecologic Oncology, Department of Obstetrics and Gynecology, University of Southern California, Los Angeles, CA USA; 5 Norris Comprehensive Cancer Center, University of Southern California, Los Angeles, CA USA; 6 Department of Obstetrics and Gynecology, Tohoku Medical and Pharmaceutical University, Miyagi, Japan; 7 Gynecology Service, National Hospital Organization Kyushu Cancer Center, Fukuoka, Japan; 8 Department of Obstetrics and Gynecology, Keio University School of Medicine, Tokyo, Japan; 9 Department of Obstetrics and Gynecology, Tokai University, Kanagawa, Japan

**Keywords:** Cervical cancer, Surgical oncology

## Abstract

The 2018 International Federation of Gynecology and Obstetrics (FIGO) revision to the staging criteria for uterine cervical cancer adopted pathological staging for patients who underwent surgery. We investigated the correlation between clinicopathological factors and prognosis in patients with high-risk factors in accordance with the FIGO 2018 staging criteria by analyzing a real-world database of 6,192 patients who underwent radical hysterectomy at 116 institutions belonging to the Japan Gynecologic Oncology Group. A total of 1,392 patients were categorized into the high-risk group. Non-squamous cell carcinoma histology, regional lymph node metastasis, pT2 classification, and ovarian metastasis were identified as independent risk factors for mortality. Based on pathological findings, 313, 1003, and 76 patients were re-classified into FIGO 2018 stages IIB, IIIC1p, and IIIC2p, respectively. Patients with stage IIIC2p disease showed worse prognoses than those with stage IIB or IIIC1p disease. In patients with stage IIIC1p disease, overall survival was significantly better if their tumors were localized in the uterine cervix, except for single lymph node metastasis, with a 5-year overall survival rate of 91.8%. This study clarified the heterogeneity of the high-risk group and provided insights into the feasibility of upfront radical hysterectomy for a limited number of patients harboring high-risk factors.

## Introduction

Uterine cervical cancer is a widely prevalent gynecological malignancy. Although most cervical cancers can be prevented through human papilloma virus (HPV) vaccination and appropriate cancer screening, not all individuals have access to these amenities^1–4^. According to a global database, in 2020, 604,127 patients were newly diagnosed with cervical cancer and 341,831 patients died from this cancer^5^. In Japan, 2,887 patients died of the disease in 2020^6^. Thus, optimization of therapeutic strategies for advanced cervical cancer remains an important concern for both patients and clinicians.

In 2018, the International Federation of Gynecology and Obstetrics (FIGO) revised their staging system for uterine cervical cancer. The most important changes in the system are the transition from a clinical to a post-surgical pathological staging system for patients whose primary treatment involved surgery and the integration of lymph node metastasis in the staging criteria reflected in FIGO 2018 stage IIIC^7^.

Radical hysterectomy and definitive radiotherapy (RT) or concurrent chemoradiotherapy (CCRT) are the fundamental therapeutic options for uterine cervical cancer without distant metastasis^8–10^. With the development of Okabayashi’s surgery, radical hysterectomy is more frequently performed in Japan than in other countries^11–14^. The National Comprehensive Cancer Network guidelines state that radical hysterectomy is a primary treatment option for patients categorized into FIGO 2018 clinical stages IB1, IB2, and IIA1, in which the tumor diameter is ≤ 40 mm. While radical hysterectomy is also listed as an option for patients in clinical stages IB3 and IIA2, CCRT is recommended with a stronger evidence level than surgery. Radical hysterectomy is not a recommended option for patients in stages IIB, III, or IVA^9^. In contrast, the latest version of the Japan Society of Gynecologic Oncology (JSGO) guidelines recommends either radical hysterectomy or RT/CCRT as the primary treatment for patients with FIGO 2018 clinical stage IB or IIA cervical cancer regardless of tumor diameter. Although CCRT is recommended, radical hysterectomy has also been proposed as an option under particular conditions for FIGO 2018 clinical stage IIB cases. Furthermore, upfront radical hysterectomy is proposed as a primary therapeutic option to CCRT for patients in FIGO 2018 stage IIIC if their T classification is T1 or T2^10^.

Patients who undergo surgery as the primary treatment are classified as showing low, intermediate, or high risk for recurrence based on pathological findings. Adjuvant therapy is considered for patients with intermediate- or high-risk factors. Pathologically proven parametrial invasion and regional lymph node metastasis are considered high-risk factors, and adjuvant CCRT is recommended^9,10,15,16^. In contrast to low- and intermediate-risk patients, the clinicopathological features of postsurgical high-risk patients, who are positive for pathologically proven parametrial invasion and/or regional lymph node metastasis, have not been fully described as these patients are more likely to be treated with definitive RT/CCRT globally^9,17^. Another concern is that lymph node metastasis detected using clinical imaging modalities such as computed tomography (CT), magnetic resonance imaging, or positron emission tomography-CT had not been officially integrated in the cancer staging system for cervical cancer until the FIGO 2018 staging system was announced^7,18^; consequently, literature on therapeutic stratification based on lymph node metastasis in the treatment of cervical cancer is lacking. Considering the current popularity of the FIGO 2018 staging schema, we believe that it is especially important to clarify the clinicopathological features of FIGO 2018 stage IIIC population.

The Japanese Gynecologic Oncology Group (JGOG) has established a nationwide database with data from 6,192 patients who underwent radical hysterectomy. We had reported the clinicopathological features of the low- and intermediate-risk groups in the database^19,20^. This study aimed to elucidate the association between patient survival and clinicopathologic features and to investigate the potential of treatment optimization in the high-risk group by retrospectively analyzing the relevant JGOG data. Because the FIGO adopted postsurgical findings in the staging criteria for the first time in 2018^7^, this study also aimed to assess the feasibility of the current staging system and clarify the clinicopathological characteristics of each FIGO 2018 stage in the high-risk group.

## Methods

The primary data included information on 6,192 patients diagnosed with uterine cervical cancer and treated with radical hysterectomy at 116 institutions under the JGOG between January 2004 and December 2008. Patient information was collected and analyzed after obtaining ethical approval from the institutional review board (IRB) at Tottori University (ethical approval number: 1946). The need for informed consent was waived due to the nature of the retrospective surveillance under the same approval by the IRB at Tottori University. All methods were performed in accordance with the relevant guidelines and regulations. The information consisted of age, FIGO 2008 clinical stage, clinical outcomes such as disease-free survival (DFS) and overall survival (OS), pathological tumor-node-metastasis (pTNM), histological diagnosis (squamous cell carcinoma [SCC] or non-SCC), tumor diameter, pelvic lymph node metastasis status, parametrial invasion, stromal invasion to the outer half, and lymphovascular space invasion. Peritoneal cytology results were reported, where applicable. If para-aortic lymphadenectomy was performed, the presence or absence of para-aortic lymph node metastasis was also recorded.

After excluding patients who received neoadjuvant chemotherapy or had distant metastasis, patients were categorized into the high-risk group if they showed positive results for lymph node metastasis and/or parametrial invasion. Patients without information about neoadjuvant chemotherapy, pTNM staging, or clinical outcomes were excluded. We also excluded cases with conflicting data for pTNM staging and parametrial invasion or lymph node metastasis.

Survival curves were determined using the Kaplan–Meier method and statistically compared using the log-rank test. Bonferroni correction was applied for post-hoc multiple comparisons. The chi-squared test was used to compare the distribution of adjuvant therapy. Hazard ratios for the clinical outcome and each parameter were determined using univariate and multivariate Cox regression models. A two-tailed p value that was less than 0.05 was considered significant. Statistical analyses were performed using JMP® Pro 16.0.0. (JMP Statistical Discovery, Cary, NC, USA).

## Results

### Patient characteristics

Based on patient selection criteria, 1,392 patients were included in the analysis. The patient selection scheme is shown in Supplementary Fig. S1. It must be noted that all patients underwent open laparotomy because minimally invasive approaches such as laparoscopic and robot-assisted surgery were not covered by public health insurance in Japan during the study period. Table 1 summarizes the patient characteristics. The most important point in the revised 2018 FIGO staging criteria for uterine cervical cancer was the adoption of a postsurgical staging system for patients who underwent surgery. Therefore, we reclassified all cases according to the FIGO 2018 staging criteria by referring to their pathological findings. Consequently, 313, 1006, and 76 patients were reclassified into FIGO 2018 stages IIB, IIIC1p, and IIIC2p, respectively (Table 1 and Supplementary Fig. S1).

**Table 1:** Patient characteristics.

Parameter	No. of patients or range (median)	%
No. of patients	1392	
Age (year)	20–83 (49)	
Observation period (months)	0–120 (59)	
Death from any cause	295	
Disease recurrence	449	
FIGO 2018 stage		
IIB	313	22.5
IIIC1p	1003	72.1
IIIC2p	76	5.5
pT classification		
pTIa	1	0.1
pTIb	495	35.6
pT2a	159	11.4
pT2b	737	52.9
Histology		
SCC	954	68.5
nSCC	438	31.5
PLN metastasis		
Negative	315	22.6
Positive	1077	77.4
PALN metastasis		
Negative	259	18.6
Positive	76	5.5
Lymphadenectomy not performed	1057	75.9
Tumor diameter		
≤ 40 mm	867	62.3
> 40 mm	513	36.9
Unknown	12	0.9
LVSI		
Negative	194	13.9
Positive	1143	82.1
Unknown	55	4.0
Stromal invasion		
≤ 1/2	238	17.1
> 1/2	1020	73.3
Unknown	134	9.6
Ovarian metastasis		
Negative	1317	94.6
Positive	32	2.3
Unknown/preserved`	43	3.1
Peritoneal cytology		
Negative	411	29.5
Positive	51	3.7
Unknown/not examined	930	66.8
Uterine corpus invasion		
Negative	1053	75.6
Positive	321	23.1
Unknown	18	1.3
Adjuvant therapy		
CCRT	573	41.2
CT	350	25.1
RT	351	25.2
None	64	4.6
Others/unknown	54	3.9

*FIGO* The International Federation of Gynecology and Obstetrics, *HR* hazard ratio, *SCC* squamous cell carcinoma, *nSCC* non-squamous cell carcinoma, *PLN* pelvic lymph node, *LVSI* lymphovascular space invasion, *CCRT* concurrent chemoradiotherapy, *CT* chemotherapy, *RT* radiotherapy.

Most patients received adjuvant CCRT, RT, or chemotherapy. More specifically, 87.5%, 93.2%, and 92.1% of the patients who were classified into FIGO 2018 stage IIB, IIIC1p, and IIIC2p, respectively, received either adjuvant CCRT, RT, or chemotherapy. Because adjuvant chemotherapy was administered more frequently to patients with non-SCC histology for intermediate-risk factors^20^, we investigated whether histological differences were statistically associated with the type of adjuvant therapy in the high-risk group. As shown in Supplementary Table S1, chemotherapy was administered to 19.6% (175/891) and 45.7% (175/383) of the patients with SCC and non-SCC histology, respectively. The chi-square test indicated significant differences in the selection of adjuvant chemotherapy between the SCC and non-SCC histology groups (p < 0.001). We also assessed the association between types of adjuvant therapy and peritoneal cytology, as positive peritoneal cytology indicates the possibility of microscopic metastasis within peritoneal cavity, which may motivate clinicians to select adjuvant chemotherapy. As shown in Supplementary Table S2, no significant difference was observed in the results of the chi-square test (*p *= 0.468).

### Comparison of survival according to FIGO 2018 stage and patterns of positive risk factors

**Figure 1:**
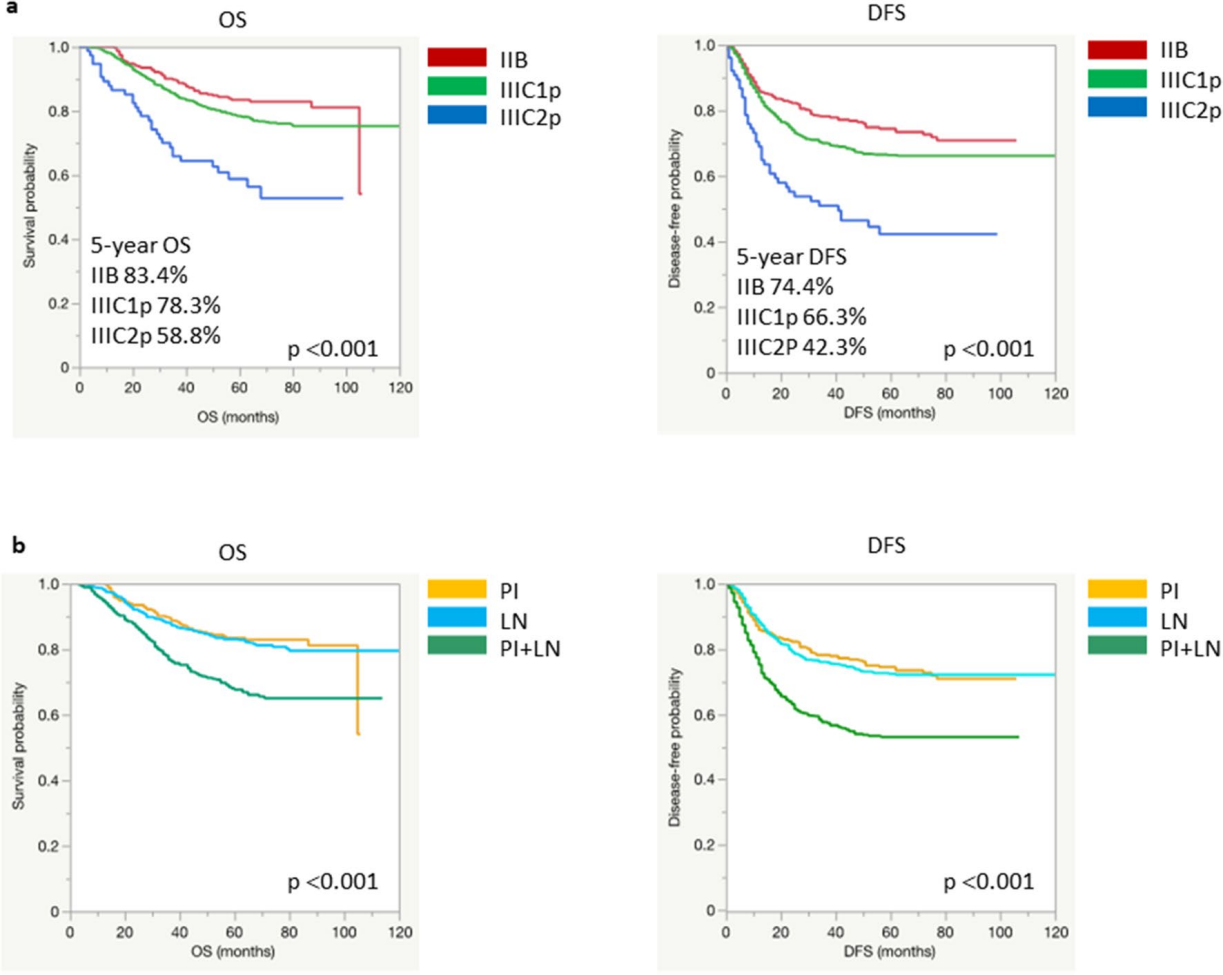
Comparison of survival by FIGO 2018 stage and by the pattern of positive risk factors. (**a**) Kaplan–Meier curves for each FIGO 2018 stage. (**b**) Kaplan–Meier curves according to the pattern of positive risk factors. *FIGO* The International Federation of Gynecology and Obstetrics, *OS* overall survival, *DFS* disease-free survival, *PI* parametrial invasion, *LN* lymph node metastasis.

To assess the feasibility of the FIGO 2018 staging criteria, we determined survival curves according to FIGO 2018 stage (Fig. 1a). While patients in stages IIB and IIIC1p showed similar survival trends, those in stage IIIC2p showed poor OS and DFS. Post-hoc analysis revealed a significant difference in both OS and DFS between stages IIIC2p and IIB or IIIC1 (Supplementary Fig. S2). In terms of risk factors, 654 patients were diagnosed with only pelvic and/or para-aortic lymph node metastasis, 313 patients were diagnosed with only parametrial invasion, and 425 patients were diagnosed with both high-risk factors. To understand the association between risk factors and prognosis, we compared survival curves on the basis of the pattern of positive risk factors. Although patients showing either high-risk factor showed similar OS and progression-free survival (PFS) trends, the OS and PFS of those exhibiting both risk factors were significantly worse, as shown in Fig. 1b.

### Association between overall survival and pathological factors

To further assess the influence of pathological factors on patient mortality, we performed univariate and multivariate Cox regression analyses. The results of univariate analysis indicated that all the pathological factors were associated with OS (Table 2). Since peritoneal cytological evaluation was not performed in over half of the 1,392 patients included in this study, peritoneal cytology data were excluded from subsequent multivariate analyses. Non-SCC, pT classification, pelvic or para-aortic lymph node metastasis, and ovarian metastasis were identified as independent risk factors for OS (Table 2). Importantly, tumor diameter, depth of stromal invasion, and lymphovascular space invasion (LVSI) were not significantly associated with the OS of high-risk patients. LVSI was identified as an independent risk factor for disease recurrence, in addition to non-SCC, pT classification, pelvic or para-aortic lymph node metastasis, and ovarian metastasis. Patients who did not receive adjuvant therapy were also at risk of recurrence (Supplementary Table S3).

**Table 2:** Univariate and multivariate analysis of overall survival for the 1,392 patients.

Variables	Univariate			Multivariate		
	HR	95% CI	*p* value	HR	95% CI	*p* value
Age (continuous)	0.992	0.983–1.002		0.988	0.977–1.000	
Histology						
SCC	1 (reference)			1 (reference)		
nSCC	2.060	1.638–2.591	< 0.001	2.381	1.814–3.127	< 0.001
pT classification						
pT1a	NA	NA	NA	NA	NA	NA
pT1b	1 (reference)			1 (reference)		
pT2a	2.085	1.421–3.061	< 0.001	2.181	1.401–3.396	< 0.001
pT2b	1.881	1.426–2.480	< 0.001	2.332	1.621–3.356	< 0.001
pT2a	1 (reference)			1 (reference)		
pT2b	0.902	0.645–1.261	0.546	1.069	0.712–1.606	0.746
PLN metastasis						
Negative	1 (reference)			1 (reference)		
Positive	1.483	1.097–2.005	0.011	1.997	1.366–2.920	< 0.001
PALN metastasis						
Negative/not performed	1 (reference)			1 (reference)		
Positive	2.474	1.705–3.590	< 0.001	1.668	1.017–2.737	0.043
Tumor diameter						
≤ 40 mm	1 (reference)			1 (reference)		
> 40 mm	1.629	1.293–2.052	< 0.001	1.283	0.978–1.683	0.072
LVSI						
Negative	1 (reference)			1 (reference)		
Positive	1.977	1.290–3.029	0.002	1.336	0.827–2.157	0.237
Stromal invasion						
≤ 1/2	1 (reference)			1 (reference)		
> 1/2	2.122	1.425–3.161	< 0.001	1.367	0.856–2.183	0.190
Ovarian metastasis						
Negative/preserved	1 (reference)			1 (reference)		
Positive	3.682	2.253–6.019	< 0.001	2.254	1.232–4.122	< 0.001
Corpus invasion						
Negative	1 (reference)			1 (reference)		
Positive	1.709	1.337–2.185	< 0.001	1.266	0.935–1.715	0.127
Adjuvant therapy						
CCRT	1 (reference)			1 (reference)		
CT	1.071	0.804–1.427	0.637	0.837	0.596–1.176	0.306
RT	1.024	0.766–1.368	0.875	1.119	0.806–1.555	0.501
None	0.884	0.427–1.685	0.707	1.120	0.504–2.487	0.781
CT	1 (reference)			1 (reference)		
RT	0.955	0.694–1.315	0.779	1.337	0.917–1.948	0.131
None	0.825	0.427–1.594	0.566	1.337	0.591–3.025	0.485
RT	1 (reference)			1 (reference)		
None	0.863	0.446–1.671	0.863	1.000	0.443–2.260	0.999
Peritoneal cytology						
Negative	1 (reference)					
Positive	2.126	1.327–3.405	0.002			

*HR* Hazard ratio,* CI* confidence interval, *SCC* squamous cell carcinoma, *nSCC* non-squamous cell carcinoma, *PLN* pelvic lymph node, *PALN* para-aortic lymph node, *LVSI* lymphovascular space invasion, *CCRT* concurrent chemoradiotherapy, *CT* chemotherapy, *RT* radiotherapy.

### Assessments of clinicopathological findings and patient survival by FIGO 2018 stage

Based on the FIGO 2018 staging criteria, the most high-risk patients are classified into stage IIB, IIIC1p, or IIIC2p^7^. To further elucidate the characteristics of each stage, we evaluated the association of clinicopathological factors and OS in stages IIB, IIIC1p, and IIIC2p independently.

The results for each substage are summarized in Tables 3, 4 and 5. In FIGO 2018 stage IIB, non-SCC histology and tumor diameter > 40 mm were identified as independent risk factors for survival from the results of the multivariate analysis (Table 3). For stage IIIC1p, we first focused on the number of lymph node metastases. Although the number of lymph node metastases and OS did not show a clear correlation (Supplementary Fig. S3), patients with multiple pelvic lymph node metastases showed significantly worse OS than those with a single lymph node metastasis (Supplementary Fig. S3). Multivariate analysis further validated the independent influence of multiple lymph node metastases on patient survival, in addition to non-SCC histology, pT2 classification, corpus invasion, and ovarian metastasis (Table 4). Because most of the risk factors identified via multivariate analysis were related to extra-cervical tumor extension, we further compared the survival between patients without extra-cervical lesions except for single lymph node involvement and the rest of the patients in stage IIIC1p. Patients whose tumors were limited to the uterine cervix and a single lymph node showed significantly better OS, with a 5-year overall survival rate of 91.8% (Supplementary Fig. S3).

**Table 3:** Univariate and multivariate analysis of overall survival in the patients classified into FIGO 2018 stage IIB.

Variables	Univariate			Multivariate		
(No. of patients)	HR	95% CI	*p* value	HR	95% CI	*p* value
Age (continuous)	0.990	0.967–1.013		0.992	0.967–1.017	
Histology						
SCC (211)	1 (reference)			1 (reference)		
nSCC (102)	2.399	1.385–4.156	0.002	3.402	1.743–6.637	< 0.001
Tumor diameter						
≤ 40 mm (194)	1 (reference)			1 (reference)		
> 40 mm (116)	2.548	1.452–4.471	0.001	2.988	1.599–5.584	< 0.001
LVSI						
Negative (60)	1 (reference)			1 (reference)		
Positive (241)	1.028	0.497–2.127	0.940	1.017	0.453–2.283	0.967
Ovarian metastasis						
Negative/preserved (307)	1 (reference)			1 (reference)		
Positive (3)	6.425	1.556–26.522	0.010	2.935	0.621–13.868	0.174
Corpus invasion						
Negative (211)	1 (reference)			1 (reference)		
Positive (102)	1.051	0.585–1.887	0.867	0.701	0.360–1.367	0.297
Adjuvant therapy						
CCRT (118)	1 (reference)			1 (reference)		
CT (57)	0.906	0.394–2.084	0.816	0.680	0.285–1.623	0.385
RT (99)	1.437	0.765–2.697	0.259	1.490	0.727–3.055	0.276
None (31)	0.431	0.100–1.858	0.259	0.382	0.084–1.730	0.212
CT	1 (reference)			1 (reference)		
RT	1.585	0.702–3.581	0.267	2.191	0.889–5.399	0.088
None	0.476	0.101–2.240	0.347	0.561	0.116–2.702	0.471
RT	1 (reference)			1 (reference)		
None	0.300	0.070–1.280	0.104	0.256	0.056–1.181	0.081

*FIGO* The International Federation of Gynecology and Obstetrics, *HR* hazard ratio, *CI* confidence interval, *SCC* squamous cell carcinoma, *nSCC* non-squamous cell carcinoma, *LVSI* lymphovascular space invasion, *CCRT* concurrent chemoradiotherapy, *CT* chemotherapy, *RT* radiotherapy.

**Table 4:** Univariate and multivariate analysis of overall survival in the patients classified into FIGO 2018 stage IIIC1p.

Variables	Univariate			Multivariate		
(No. of patients)	HR	95% CI	*p* value	HR	95% CI	*p* value
Age (continuous)	0.995	0.983–1.006		0.989	0.976–1.003	
Histology						
SCC (697)	1 (reference)			1 (reference)		
nSCC (306)	2.019	1.540–2.645	< 0.001	2.157	1.560–2.983	< 0.001
pT classificaion						
T1a (1)	NA	NA	NA	NA	NA	NA
T1b (474)	1 (reference)			1 (reference)		
T2a (149)	2.165	1.452–3.228	< 0.001	2.158	1.355–3.436	0.001
T2b (379)	2.359	1.731–3.214	< 0.001	2.367	1.594–3.515	< 0.001
T2a	1 (reference)			1 (reference)		
T2b	1.089	0.756–1.569	0.645	1.097	0.715–1.684	0.672
Tumor diameter						
≤ 40 mm (629)	1 (reference)			1 (reference)		
> 40 mm (366)	1.384	1.053–1.819	0.020	1.034	0.750–1.425	0.840
LVSI						
Negative (130)	1 (reference)			1 (reference)		
Positive (834)	2.222	1.291–3.824	0.004	1.411	0.767–2.596	0.268
Stromal invasion						
≤ 1/2 (200)	1 (reference)			1 (reference)		
> 1/2 (701)	2.065	1.346–3.169	< 0.001	1.134	0.687–1.874	0.622
No. of positive PLN metastasis						
Single (424)	1 (reference)			1 (reference)		
Multiple (574)	2.626	1.912–3.607	< 0.001	2.089	1.458–2.994	< 0.001
Ovarian metastasis						
Negative/preserved (945)	1 (reference)			1 (reference)		
Positive (18)	3.663	1.937–6.928	< 0.001	2.377	1.193–4.735	0.014
Corpus invasion						
Negative (793)	1 (reference)			1 (reference)		
Positive (192)	2.022	1.506–2.715	< 0.001	1.440	1.002–2.069	0.049
Adjuvant therapy						
CCRT (432)	1 (reference)			1 (reference)		
CT (261)	1.075	0.777–1.487	0.664	0.940	0.643–1.375	0.751
RT (242)	0.831	0.580–1.189	0.311	0.969	0.651–1.443	0.879
None (31)	1.407	0.652–3.033	0.384	1.855	0.661–5.296	0.241
CT	1 (reference)			1 (reference)		
RT	0.773	0.524–1.141	0.195	1.031	0.659–1.612	0.894
None	1.309	0.598–2.864	0.500	1.973	0.692–5.619	0.203
RT	1 (reference)			1 (reference)		
None	1.693	0.762–3.762	0.196	1.913	0.663–5.518	0.230
Peritoneal cytology						
Negative (277)	1 (reference)					
Positive (41)	1.914	1.092–3.355	0.023			

*FIGO* The International Federation of Gynecology and Obstetrics, *HR* hazard ratio, *CI* confidence interval, *SCC* squamous cell carcinoma, *nSCC* non-squamous cell carcinoma, *PLN* pelvic lymph node, *LVSI* lymphovascular space invasion, *CCRT* concurrent chemoradiotherapy, *CT* chemotherapy, *RT* radiotherapy.

**Table 5:** Univariate and multivariate analysis of overall survival in the patients classified into FIGO 2018 stage IIIC2p.

Variables	Univariate			Multivariate		
(No. of patients)	HR	95% CI	*p* value	HR	95% CI	*p* value
Age (continuous)	0.986	0.957–1.017		0.959	0.909–1.009	
Histology						
SCC (46)	1 (reference)			1 (reference)		
nSCC (30)	1.017	0.790–3.269	0.191	1.303	0.412–4.125	0.652
pT classificaion						
T1b (21)	1 (reference)			1 (reference)		
T2a (10)	1.261	0.315–5.048	0.743	0.872	0.135–5.615	0.885
T2b (45)	2.344	0.946–5.806	0.066	1.497	0.345–6.498	0.590
T2a	1 (reference)			1 (reference)		
T2b	1.859	0.556–6.216	0.314	1.717	0.342–8.611	0.511
Tumor diameter						
≤ 40 mm (44)	1 (reference)			1 (reference)		
> 40 mm (31)	2.178	1.057–4.488	0.035	1.881	0.541–6.535	0.320
Stromal invasion						
≤ 1/2 (7)	1 (reference)			1 (reference)		
> 1/2 (57)	2.699	0.364–19.996	0.331	0.841	0.095–7.479	0.877
Ovarian metastasis						
Negative/preserved (65)	1 (reference)			1 (reference)		
Positive (11)	1.447	0.554–3.777	0.451	0.611	0.066–5.678	0.665
Corpus invasion						
Negative (49)	1 (reference)			1 (reference)		
Positive (27)	1.631	0.803–3.314	0.176	1.394	0.441–4.400	0.572
Adjuvant therapy						
CCRT (26)	1 (reference)			1 (reference)		
CT (34)	0.875	0.337–2.270	0.783	0.908	0.220–3.738	0.893
RT (10)	5.707	2.112–15.423	< 0.001	5.694	1.346–24.084	0.018
None (2)	10.324	1.179–90.391	0.035	31.191	1.509–644.748	0.026
CT	1 (reference)			1 (reference)		
RT	6.526	2.506–16.999	< 0.001	6.274	1.751–22.485	0.018
None	11.805	1.373–101.479	0.025	34.369	2.073–569.752	0.014
RT	1 (reference)			1 (reference)		
None	1.809	0.218–14.981	0.583	5.478	0.299–100.284	0.252
Peritoneal cytology						
Negative (30)	1 (reference)					
Positive (9)	1.452	0.555–3.794	0.447			
LVSI						
Negative (4)	1 (reference)					
Positive (68)	NA	NA	NA			
PLN metastasis						
Negative (2)	1 (reference)					
Positive (74)	NA	NA	NA			

*FIGO* The International Federation of Gynecology and Obstetrics, *HR* hazard ratio, *CI* confidence interval, *SCC* squamous cell carcinoma, *nSCC* non-squamous cell carcinoma, *PLN* pelvic lymph node, *LVSI* lymphovascular space invasion, *CCRT* concurrent chemoradiotherapy, *CT* chemotherapy, *RT* radiotherapy.

Among the 76 FIGO 2018 IIIC2p cases, neither pathological factor was significantly associated with patient survival in a multivariate analysis. However, patients treated with adjuvant RT showed significantly worse OS than those treated with adjuvant CCRT or chemotherapy, even though the number of patients who received each adjuvant therapy was relatively small (Table 5).

## Discussion

In this study, we investigated the association between patient prognosis and clinicopathological features in surgically treated patients with uterine cervical cancer harboring high-risk factors. Overall, the results indicated that patient prognosis was heterogeneous, even in the high-risk group.

As shown in Table 2, non-SCC histology, pT2a/pT2b classification, pelvic or para-aortic lymph node metastasis, and ovarian metastasis were identified as independent risk factors for OS in the high-risk group. In contrast, LVSI, deep stromal invasion, and tumor diameter, which have been categorized as intermediate-risk factors^21,22^, did not influence OS. These results validate the significance of parametrial invasion and lymph node metastasis in patient prognosis. Simultaneously, non-SCC histology and ovarian metastasis were identified as potential risk factors among the high-risk groups. Because pT2a represents vaginal invasion, pathological vaginal invasion may be another risk factor. Importantly, these factors were also identified as risk factors for mortality in the low- and intermediate-risk groups^19,20^. Our findings suggest that, in addition to conventional risk factors, other pathological parameters that are not currently deemed indisputable risk factors should be considered when planning therapeutic strategies in patients with surgically treated cervical cancer.

Adherence to the guidelines regarding adjuvant therapy for patients primarily treated with radical hysterectomy is an unresolved issue in Japan. Although the current Japanese guidelines recommend adjuvant CCRT for treating high-risk patients^10^, adjuvant chemotherapy was adopted in approximately a quarter of the patients, especially in the non-SCC population. One reason for this may be to avoid radiation-related adverse events^23,24^. Indeed, Ikeda et al. reported that an increasing proportion of patients were treated with adjuvant chemotherapy in Japan^25^. Moreover, radiation has been considered to be less effective for adenocarcinoma in several studies, which may lead physicians to prescribe adjuvant chemotherapy for non-SCC cervical cancer^12,26–28^. Nevertheless, evidence for the feasibility of adjuvant chemotherapy in high-risk groups is currently inadequate. Unfortunately, we estimate that our database had substantial physician and institutional biases, making it difficult to appropriately assess the influence of adjuvant therapy. In the light of the current situation, the JGOG is conducting a prospective study to compare adjuvant CCRT and chemotherapy^29^. The results of this study will provide a certain indication regarding this concern.

Another concern is related to surgical radicality and the site of recurrence. A study by Matsuo et al. reported a lower risk of distant metastasis and a higher risk of local recurrence among patients with node-positive FIGO2008 stage IB-IIB cervical cancer who received adjuvant chemotherapy, which indicates that a satisfactory local control rate should be achieved through radical hysterectomy if adjuvant chemotherapy is selected^30^.

In classifications of cervical cancer based on the FIGO 2018 staging criteria, stages IIB, IIIC1p, and IIIC2p corresponded to the most high-risk cases. We believe that the inclusion of cases in which para-aortic lymph nodes were pathologically examined is an advantage of the current investigation of the feasibility of the FIGO 2018 staging criteria. Our analysis supported the feasibility of FIGO 2018 IIB, IIIC1p, and IIIC2p classifications from the perspective of prognosis. Patients categorized into stage IIIC2p showed significantly worse prognosis than those classified into stages IIB and IIIC1p. Interestingly, adjuvant RT was indicated as a risk factor for mortality compared with adjuvant CCRT and CT. Despite the previously mentioned limitations in the assessment of adjuvant therapy, our findings highlight the necessity of intensive adjuvant therapy for surgically treated patients with FIGO 2018 stage IIIC2p disease. Moreover, the prognosis observed in this study raised a concern regarding the feasibility of surgery-based therapeutic strategies in patients presenting with clear para-aortic lymph node metastasis as indicated by clinical imaging.

Patients with FIGO2018 IIIC1p cervical cancer showed heterogeneous prognoses based on the number of lymph node metastases and status of tumor extension. While definitive CCRT is generally considered if lymph node metastasis is suspected on preoperative clinical imaging, our results indicated that a patient was likely to have a favorable prognosis with the upfront surgery approach if the tumor was estimated to be limited to the uterine cervix except for single lymph node involvement. This finding is concordant with previous retrospective studies that reported the number of lymph node metastases as a risk factor for patients with stage IIIC1p cervical cancer^31,32^. On the other hand, the treatment strategies for patients with suspected multiple lymph node metastasis or tumor extension beyond uterine cervix should be optimized for each patient based on performance status, preexisting comorbidities, expected adverse effects, cost-effectiveness, and so on, along with careful preoperative physical evaluation and multiple clinical imaging.

One limitation of this study is that the current database consisted of information collected only from surgically treated patients. Therefore, direct comparison of treatment outcomes between primary radical hysterectomy with subsequent adjuvant therapy and definitive CCRT was not possible. Not limited to patients treated with upfront surgery, the prognosis of patients with FIGO2018 IIIC1 disease was reported to be heterogeneous depending on local tumor factors in a retrospective cohort study^33^. On the other hand, a nationwide clinicopathological database containing information on patients with uterine cervical cancer harboring high-risk factors is of significant value because these patients are more likely to be treated with RT/CCRT globally. Our findings and the existing literature highlight the need for larger-scale and more inclusive surveillance to optimize the management of FIGO 2018 stage IIIC1 cases.

In conclusion, this study clarified the heterogeneous outcomes associated with the clinicopathological features of patients presenting high-risk factors who underwent radical hysterectomy. The results reinforce the need for optimizing therapeutic strategies for this population, which should be further investigated in future studies.

## Supplementary Information

Supplementary Information.

## Data Availability

The dataset analyzed in this study is available from the corresponding author on reasonable request.
