# Supplementary material for: Surgically treated cervical cancer in a high-risk group in the era of the 2018 FIGO staging schema: a nationwide study
Source: Sci Rep. 2023 Jul 25;13:12020. doi: 10.1038/s41598-023-39014-8 (PMC10368631; doi:10.1038/s41598-023-39014-8)
Supplement: Supplementary file 1 — Supplementary Information. [file 41598_2023_39014_MOESM1_ESM.docx]

**Surgically treated cervical cancer in a high-risk group in the era of the 2018 FIGO staging schema: A nationwide study**

Shogo Shigeta, Muneaki Shimada, Keita Tsuji, Zen Watanabe, Yasuhito Tanase, Koji Matsuo, Toru Nakanishi, Toshiaki Saito, Daisuke Aoki, Mikio Mikami

**Supplementary Fig S1. Patient selection flowchart**


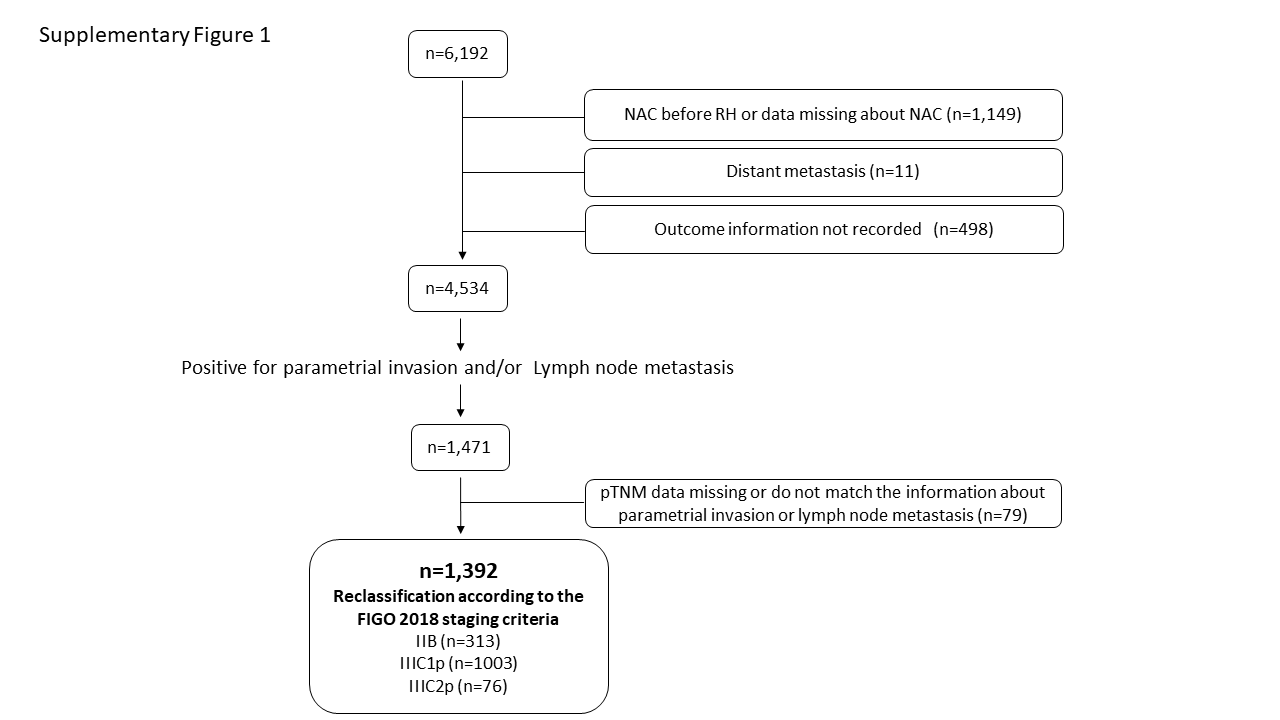


The result of patient selection is summarized in this flowchart.

Abbreviations: RH, radical hysterectomy; NAC, neoadjuvant chemotherapy; pTNM, pathological tumor-node-metastasis; FIGO, The International Federation of Gynecology and Obstetrics

**Supplementary Fig S2. Post-hoc analyses of survival comparison by FIGO 2018 stage**


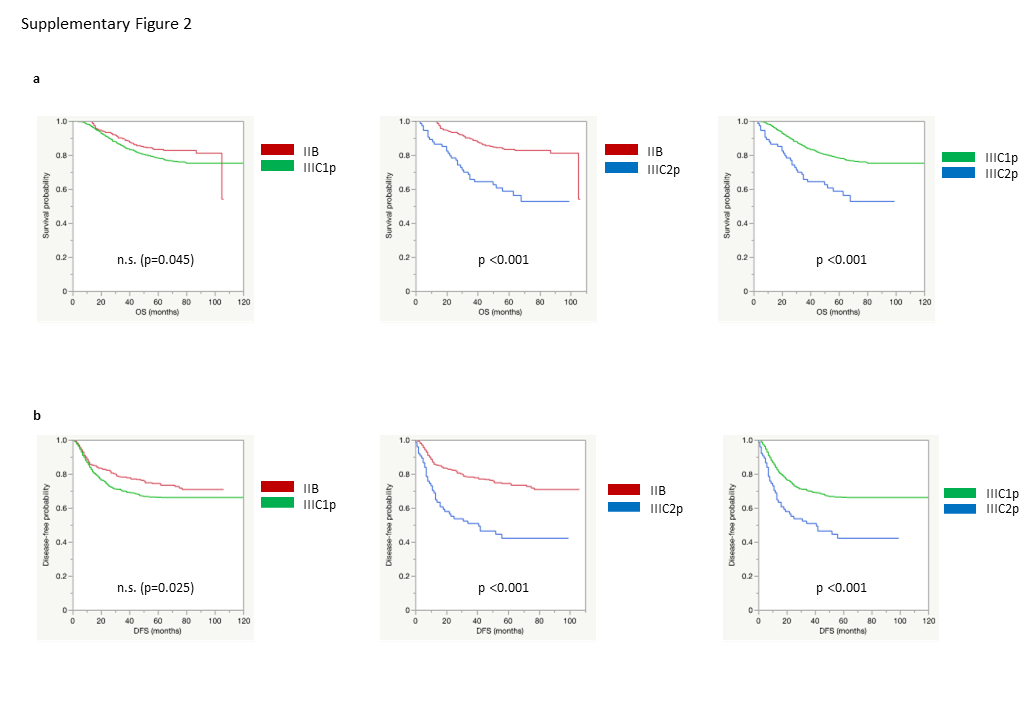


1. Post-hoc analyses of overall survival comparison by FIGO 2018 stage.
2. Post-hoc analyses of disease-free survival comparison by FIGO 2018 stage.

Abbreviations: FIGO, The International Federation of Gynecology and Obstetrics; OS, overall survival; DFS, disease-free survival; PI, parametrial invasion; LN, lymph node metastasis.

**Supplementary Fig S3. Association between patient survival and the number of lymph node metastases**


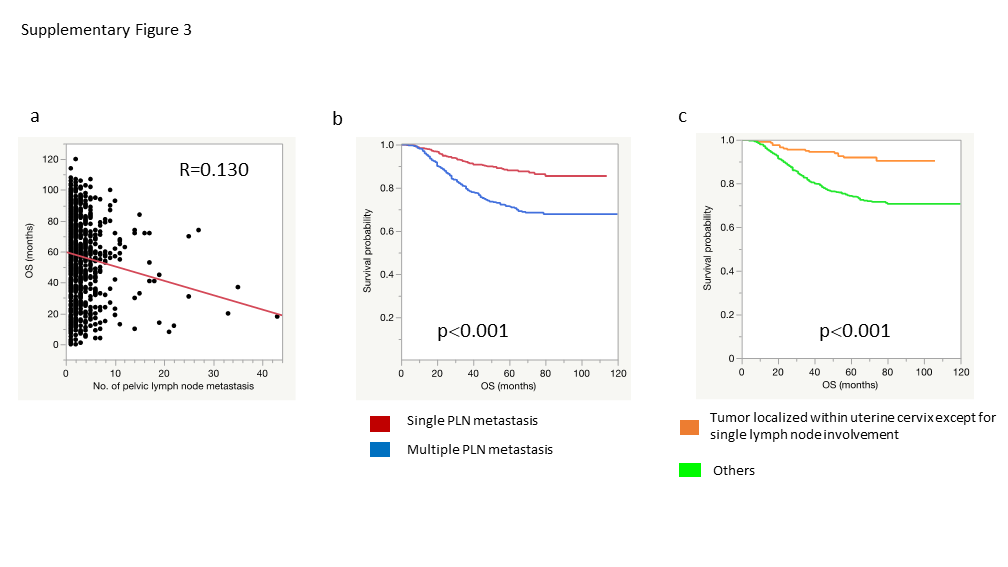


1. A scatter plot depicting the correlation between the number of positive pelvic lymph node metastases and overall survival in FIGO stage IIIC1p disease. R: correlation coefficient.
2. Comparison of survival between single and multiple lymph node-positive cases of FIGO 2018 stage IIIC1p disease.
3. Comparison of survival between patients with and without extra-cervical lesions, except for those with single lymph node metastasis.

Abbreviations: FIGO, The International Federation of Gynecology and Obstetrics; PLN, pelvic node metastasis.

**Supplementary Table S1. Two-by-two comparisons of the types of adjuvant therapy and histology**

|  | SCC | non-SCC | Total |
| --- | --- | --- | --- |
| CCRT/RT | 716 | 208 | 924 |
| CT | 175 | 175 | 350 |
| Total | 891 | 383 | 1274 |

Abbreviations: SCC, squamous cell carcinoma; CCRT, concurrent chemoradiotherapy; RT, radiotherapy; CT, chemotherapy.

**Supplementary Table S2. Two-by-two comparisons of the types of adjuvant therapy and peritoneal cytology.**

|  | PC-positive | PC-negative | Total |
| --- | --- | --- | --- |
| CCRT/RT | 29 | 259 | 288 |
| CT | 17 | 120 | 137 |
| Total | 46 | 379 | 425 |

Abbreviations: PC, peritoneal cytology; CCRT, concurrent chemoradiotherapy; RT, radiotherapy; CT, chemotherapy.

**Supplementary Table S3. Univariate and multivariate analyses of disease-free survival in the 1,392 patients**

| **Variables** | | **Univariate** | | |  | **Multivariate** | | |
| --- | --- | --- | --- | --- | --- | --- | --- | --- |
|  |  | **HR** | **95% CI** | **p value** |  | **HR** | **95% CI** | **p value** |
| **Age (continuous)** |  | 0.996 | 0.988-1.004 |  |  | 0.991 | 0.982-1.000 |  |
| **Histology** |  |  |  |  |  |  |  |  |
|  | SCC | 1 (reference) |  |  |  | 1 (reference) |  |  |
|  | Non-SCC | 1.784 | 1.480-2.152 | <0.001 |  | 2.021 | 1.625-2.512 | <0.001 |
| **pT classification** |  |  |  |  |  |  |  |  |
|  | pT1a | NA | NA | NA |  | NA | NA | NA |
|  | pT1b | 1 (reference) |  |  |  | 1 (reference) |  |  |
|  | pT2a | 1.833 | 1.340-2.508 | <0.001 |  | 1.947 | 1.367-2.772 | <0.001 |
|  | pT2b | 1.803 | 1.448-2.442 | <0.001 |  | 2.348 | 1.767-3.119 | <0.001 |
|  | pT2a | 1 (reference) |  |  |  | 1 (reference) |  |  |
|  | pT2b | 0.983 | 0.744-1.300 | 0.907 |  | 1.206 | 0.865-1.681 | 0.269 |
| **PLN metastasis** |  |  |  |  |  |  |  |  |
|  | Negative | 1 (reference) |  |  |  | 1 (reference) |  |  |
|  | Positive | 1.409 | 1.109-1.791 | 0.005 |  | 1.988 | 1.475-2.678 | <0.001 |
| **PALN metastasis** |  |  |  |  |  |  |  |  |
|  | Negative/not performed | 1 (reference) |  |  |  | 1 (reference) |  |  |
|  | Positive | 2.204 | 1.598-3.040 | <0.001 |  | 1.691 | 1.123-2.544 | 0.012 |
| **Tumor diameter** |  |  |  |  |  |  |  |  |
|  | ≤40 mm | 1 (reference) |  |  |  | 1 (reference) |  |  |
|  | >40mm | 1.512 | 1.254-1.823 | <0.001 |  | 1.192 | 0.961-1.480 | 0.110 |
| **LVSI** |  |  |  |  |  |  |  |  |
|  | Negative | 1 (reference) |  |  |  | 1 (reference) |  |  |
|  | Positive | 2.567 | 1.761-3.742 | <0.001 |  | 2.052 | 1.349-3.119 | <0.001 |
| **Stromal invasion** |  |  |  |  |  |  |  |  |
|  | 1/2 | 1 (reference) |  |  |  | 1 (reference) |  |  |
|  | >1/2 | 1.917 | 1.415-2.598 | <0.001 |  | 1.333 | 0.935-1.901 | 0.113 |
| **Ovarian metastasis** |  |  |  |  |  |  |  |  |
|  | Negative/preserved | 1 (reference) |  |  |  | 1 (reference) |  |  |
|  | Positive | 3.130 | 2.036-4.812 | <0.001 |  | 2.177 | 1.282-3.698 | 0.004 |
| **Corpus invasion** |  |  |  |  |  |  |  |  |
|  | Negative | 1 (reference) |  |  |  | 1 (reference) |  |  |
|  | Positive | 1.355 | 1.101-1.667 | 0.004 |  | 0.988 | 0.769-1.271 | 0.927 |
| **Adjuvant therapy** |  |  |  |  |  |  |  |  |
|  | CCRT | 1 (reference) |  |  |  | 1 (reference) |  |  |
|  | CT | 0.933 | 0.738-1.179 | 0.560 |  | 0.768 | 0.583-1.013 | 0.062 |
|  | RT | 0.896 | 0.706-1.135 | 0.362 |  | 1.043 | 0.805-1.352 | 0.750 |
|  | None | 1.078 | 0.680-1.708 | 0.751 |  | 1.741 | 1.023-2.961 | 0.041 |
|  | CT | 1 (reference) |  |  |  | 1 (reference) |  |  |
|  | RT | 0.960 | 0.735-1.254 | 0.766 |  | 1.357 | 0.996-1.850 | 0.053 |
|  | None | 1.156 | 0.717-1.861 | 0.552 |  | 2.265 | 1.303-3.938 | 0.004 |
|  | RT | 1 (reference) |  |  |  | 1 (reference) |  |  |
|  | None | 1.203 | 0.746-1.940 | 0.448 |  | 1.669 | 0.964-2.888 | 0.067 |
| **Peritoneal cytology** |  |  |  |  |  |  |  |  |
|  | Negative | 1 (reference) |  |  |  |  |  |  |
|  | Positive | 1.969 | 1.323-2.931 | <0.001 |  |  |  |  |

Abbreviations: HR, hazard ratio; SCC, squamous cell carcinoma; PLN, pelvic lymph node; PALN, para-aortic lymph node; LVSI, lymphovascular space invasion; CCRT, concurrent chemoradiotherapy; CT, chemotherapy; RT, radiotherapy
